# Health Disparities Associated with Females Reporting Human Papillomavirus Infection in the United States

**DOI:** 10.1089/whr.2021.0036

**Published:** 2021-07-14

**Authors:** Man Hung, Sharon Su, Eric S. Hon, Frank W. Licari, Jungweon Park, Jerry Bounsanga, Jacob Tuft, Sylvia Otrusinik, Martin S. Lipsky

**Affiliations:** ^1^ Roseman University of Health Sciences College of Dental Medicine, South Jordan, Utah, USA.; ^2^ University of Utah School of Medicine, Salt Lake City, Utah, USA.; ^3^ University of Utah College of Education, Salt Lake City, Utah, USA.; ^4^ Huntsman Cancer Institute, Salt Lake City, Utah, USA.; ^5^ University of Chicago Department of Economics, Chicago, Illinois, USA.; ^6^ Portland State University Institute on Aging, Portland, Oregon, USA.

**Keywords:** HPV, females, NHANES, public health, education

## Abstract

Few studies provide detailed findings about the health disparities of women being told by a physician whether they have ever had a human papillomavirus (HPV) infection. This study sought to characterize the prevalence and characteristics associated with women age 18 to 59 years in the United States who report being told they were infected with HPV. This study used data from the National Health and Nutritional Examination Survey. Descriptive statistics were computed on study variables and multiple logistic regression analyses were conducted to explore the association of the study variables with the outcome variable. Sampling weights were applied to produce national estimates of prevalence. The sample consisted of 1,669 females, representative of 75,107,170 females in the United States population. Around 11.5% reported being told that they had an HPV infection, of which 60.9% were White, and 82.9% were born in the United States. White women are 2.0 times more likely to be told they have HPV than Asian women and 2.8 times more likely than Black women. United States-born women were 2.1 times more likely told they had an HPV infection than those foreign born. This study found that among U.S. women, less than 12% reported ever having been told they have had an HPV infection. Epidemiologic findings suggest gaps between ever being told of a previous infection and being diagnosed with a clinically relevant HPV infection. Despite epidemiologic data indicating higher HPV prevalence among those less educated and women of color, these groups were less likely to report ever being told they have an HPV infection than White women, and those with a college degree suggesting communication gaps among these subgroups about HPV infection that might exist. Strategies to address potential gaps in communication among these subgroups can potentially reduce the economic burden and health disparities related to HPV infection.

## Introduction

Human papillomavirus (HPV) is the most common sexually transmitted infection.^1,2^ Spread by skin to skin contact, HPV genital infections in women can be divided into lower risk types that may lead to genital warts and benign cervical changes or higher risk types associated with cervical and other genital cancers.^3^ While most HPV infections resolve without significant clinical consequences, HPV infections, especially high-risk subtypes 16 and 18, are the main cause of cervical cancer.^4^ Each year an estimated 13,000 women will be diagnosed with cervical cancer in the United States and more than 4,000 women will die from it.^5^ However, the incidence rates of cervical cancer are changing in part because of better screening^6^ and HPV vaccination.^7^ Trends in HPV infection in men also affect HPV prevalence in women,^8^ and the 2011 ACIP recommendation to vaccinate males 9 to 26 years of age impacts women as well as men.^9^

However, the overall prevalence in women of being infected with low and high-risk genital HPV for adults 18 to 59 years of age remains about 40% and 20%, respectively.^10^ Multiple meta-analyses investigating trends and profiles among individuals with HPV found the highest infection rates in sexually active women under the age of 25^11^ and younger women are also more likely to have abnormal cervical cytology. Other identified risk factors for HPV include a greater number of sexual partners, lower education level, lower income, smoking, and being uninsured.^12^ Prevalence is also lower among non-Hispanic Asian and higher among non-Hispanic Black women than non-Hispanic white and Hispanic.^10^

International epidemiology parallels findings seen in the United States. A 2007 global meta-analysis found that HPV was more common in women under the age of 35 and in those with abnormal cervical cytology or cervical cancer.^13^ Another meta-analysis investigating the prevalence of HPV found that HPV was most common in Africa, Eastern Europe, and Latin America in those under age 25.^14^ Both studies identified a second peak of HPV in Africa and the Americas in women age 45 years and older.

Despite an extensive body of epidemiologic HPV literature, few studies provide detailed data about the prevalence of women being told by a physician about whether they have ever had an HPV infection. While many women with HPV never develop symptoms or problems, those with clinically apparent infections or who test positive on a screening exam should know if they have been infected. Knowledge about ever being told about an HPV infection likely affects whether a woman follows recommendations regarding cervical cancer screening, adheres to treatment, and shape attitudes toward HPV immunization. An understanding of the characteristics of women who reported having been told they had an HPV infection can help providers and public health officials to identify if potential communication gaps between women experiencing a clinically relevant infection and being told they have an infection exist. Addressing these gaps can facilitate strategies to improve communication about HPV infection. This study adds to the literature by using a national database to characterize the prevalence and characteristics associated with women in the United States who report ever being told they were infected with HPV. Understanding these associations can help clinicians and public health personnel to educational awareness programs that potentially improve screening and follow-up, decrease health disparities, and increase immunization rates.

## Methods

This study used data from 2015 to 2016 National Health and Nutritional Examination Survey (NHANES) to examine the prevalence and characteristics related to women in the United States who report ever being told they had an HPV infection. NHANES uses a cross-sectional survey to monitor the health and nutritional status of the civilian noninstitutionalized United States population. The survey consists of interviews conducted in participants' homes and includes demographic, socioeconomic, dietary, and other health-related questions followed by standardized physical examinations. NHANES oversamples some populations to obtain more precise estimates for subgroups and to help assure a nationally representative sample. Data are publicly available and released in 2-year cycles. Details about NHANES design and data collection can be found at https://www.cdc.gov/nchs/nhanes/index.htm.

Included in the analyses of the 2015–2016 NHANES data were all females age 18 to 59 years that responded on the variable “Ever told by MD you had HPV.” Descriptive statistics were computed on study variables (*i.e.*, race, marital status, income, education, country of birth, citizenship, whether or not they received the HPV vaccine, and whether or not they have been told by MD that they had genital warts), and presented graphically. Cases were weighted by the full-sample 2-year interview weight for demographic characteristics computation to produce nationally representative estimates. For statistical tests, unweighted values of the sample were used. Chi-square tests were conducted on the association of the study variables with the outcome variable (*i.e.*, whether an individual reported ever being told they had an HPV infection). Multiple logistic regression analyses were also performed to assess the independent effect of the study variables with the outcome. Statistical significance was set at alpha of 0.05 and statistical analyses were performed using SPSS 26. The National Center for Health Statistics Institutional Review Board reviewed and approved NHANES, and participants provided written informed consent.

## Results

The study consisted of 75,107,170 females (unweighted *n* = 1,669) representative of the United States population. Their age ranged from 18 to 59 years, of which 60.9% was White, 13.0% Black, and 10.3% Mexican American. The average age was 39.4 years (standard deviation = 11.9) and majority were born in the United States (85.9%) (Table 1). The proportion of the females in the weighted sample representing the United States women who reported having ever been told they were diagnosed with HPV was 11.5% (Table 1). For those who had genital warts, the proportion that did not report HPV (56.7%) was significantly higher than the proportion that reported HPV (43.3%) (*p* < 0.05).

**Table 1.** Demographic Characteristics (Unweighted *n* = 1,669; Weighted *N* = 75,107,170)

Variable	Mean (SD)^a^	Mean (SD)^b^	*n* (%)^a^	*N* (%)^b^
Age in years	38.41 (11.96)	39.40 (11.90)		
Race/ethnicity				
Mexican American			321 (19.2)	7,704,969 (10.3)
Other Hispanic			218 (13.1)	5,124,600 (6.8)
Non-Hispanic White			500 (30.0)	45,747,189 (60.9)
Non-Hispanic Black			397 (23.8)	9,763,877 (13.0)
Non-Hispanic Asian			164 (9.8)	3,686,930 (4.9)
Other Race			69 (4.1)	3,079,605 (4.1)
Marital status				
Married			748 (47.3)	38,931,772 (53.1)
Widowed			32 (2.0)	1,651,390 (2.3)
Divorced			175 (11.1)	7,436,631 (10.1)
Separated			74 (4.7)	2,534,041 (3.5)
Never married			360 (22.8)	14,179,446 (19.3)
Living with partner			192 (12.1)	8,607,904 (11.7)
Country of birth				
Born in the United States			1,168 (70.0)	62,271,256 (85.9)
Born outside of the United States			501 (30.0)	12,835,913 (17.1)
Received HPV vaccine				
Yes			618 (22.1)	20,841,324 (20.0)
No			2,182 (77.9)	83,242,062 (80.0)
Being told by MD that they had genital warts				
Yes			67 (4.0)	4,186,025 (5.7)
No			1,604 (96.0)	68,897,830 (94.3)
Being told by MD that they had HPV				
Yes			140 (8.4)	8,628,073 (11.5)
No			1,529 (91.6)	66,479,097 (88.5)

^a^ Unweighted estimates.

^b^ Weighted estimates.

HPV, human papillomavirus; SD, standard deviation.

**FIG. 1.**
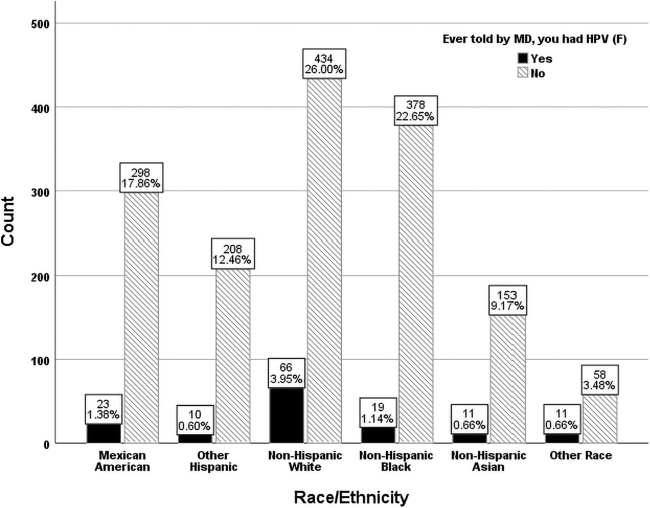
Racial distribution of females with and without HPV (unweighted *n* = 1,669). HPV, human papillomavirus.

Among the sample group, Figure 1 shows that Whites (3.95%) represented the largest subgroup of women who reported being diagnosed with HPV, followed by Mexican Americans (1.38%), Blacks (1.14%), and Asians (0.66%). Overall, 8.39% of the sample reported being told that they had an HPV infection. Among the sample who had even been told that had an HPV infection, 47.1% were Whites, 16.4% Mexican American, 13.6% of Blacks, and 7.9% Asians. White women were 2.0 times more likely to be told they have HPV than Asian women and 2.8 times more likely than Black women.

**FIG. 2.**
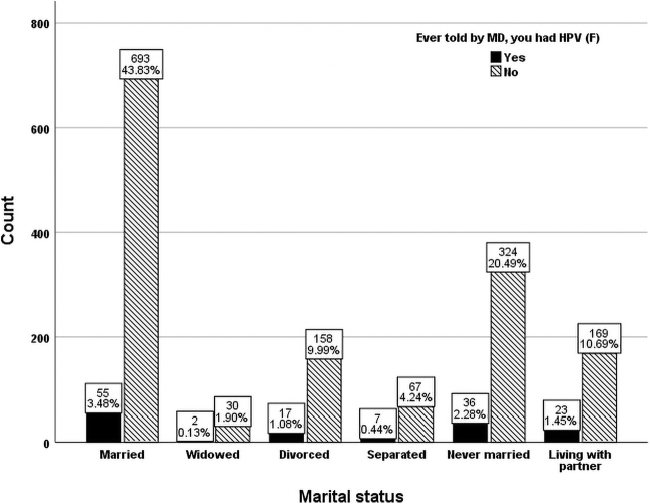
Marital distribution of females with and without HPV (unweighted *n* = 1,581).

**FIG. 3.**
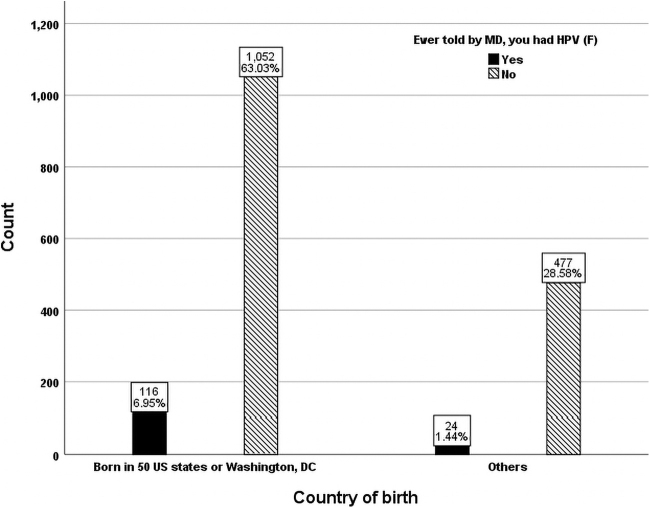
Country of birth distribution of females with and without HPV (unweighted *n* = 1,669).

In terms of marital status, married women said they were diagnosed with HPV more often than women in other categories (Fig. 2). Divorced women or those living with a partner reported lower HPV infection rates and those widowed had the lowest percentage of being told they had HPV. Females living with a partner (12.0%) who were diagnosed with HPV infection was significantly higher (odds ratio = 0.558; 95% confidence interval = [0.319–0.977]; *p* < 0.05) than those married (7.4%) even after adjusting for age, race, ethnicity, marital status, educational level, and citizenship. Approximately 70% of the sample were born in the United States and 30% were born in other countries (Fig. 3). United States-born women were 2.1 times more likely told they had an HPV infection than those foreign born.

**FIG. 4.**
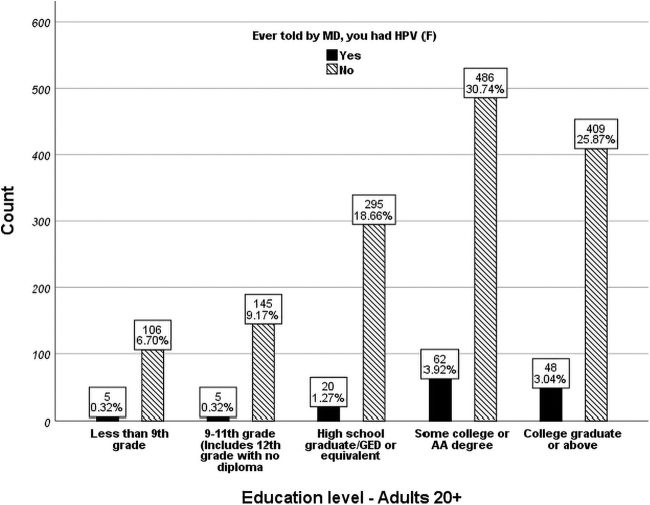
Education status distribution of females with and without HPV (unweighted *n* = 1,581).

**FIG. 5.**
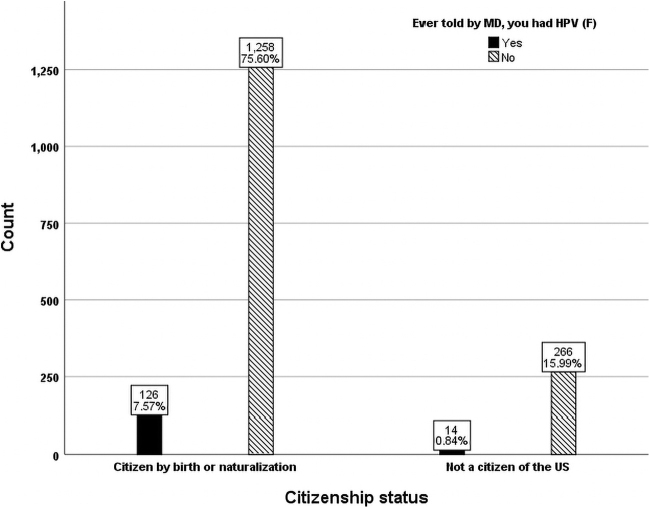
Citizenship status distribution of females with and without HPV (unweighted *n* = 1,664).

Race and ethnicity also emerged as independent variables in predicting reporting an HPV infection after adjusting for all other factors. Mexican American women had 157% greater odds of reporting HPV than Black women (odds ratio = 2.573; 95% confidence interval = [1.288–4.998]; *p* < 0.05) (Fig. 4). The “other race” category exhibited a 181% greater odds of reporting HPV than White women (odds ratio = 2.828; 95% confidence interval = [1.097–7.287]; *p* < 0.05), and 286% greater odds than Asian (odds ratio = 3.861; 95% confidence interval = [1.710–8.720]; *p* < 0.05 (Fig. 4). Those with college degree or above had 87% greater odds having been told they had an HPV infection than those with a high school diploma or equivalent (odds ratio = 1.871; 95% confidence interval = [1.058–3.309]; *p* < 0.05), and 259% greater odds than those with some high school education but without high school diploma (odds ratio = 3.592; 95% confidence interval = [1.349–9.563]; *p* < 0.05). In general, college educated women were 2.9 times more likely to be told they had an HPV infection than women that never completed high school. Country of citizenship also affected the prevalence of reporting an HPV infection with 9.1% of United States citizens reporting yes to ever having an infection compared with 5.0% of non-United States citizens (Fig. 5).

## Discussion

This study found that in a nationally representative study sample of United States women 18 to 59 years of age, less than 12% of United States women reported ever being told they had HPV. Knowledge of a previous infection correlates with HPV health literacy and knowledge.^15^ Health literacy links to improved adherence for both acute and chronic disease,^16^ making it important that women know if they have ever had an HPV infection.

Ideally women reporting ever having an infection should be a proxy for prevalence of a symptomatic HPV infection or a positive screening test. However, epidemiologic findings suggest that there are gaps between women experiencing a clinically relevant or symptomatic HPV infection and women reporting ever being told they have an HPV infection. In 2013–2014, the prevalence of any and high-risk genital HPV for adults 18 to 59 years of age was 45.2% and 25.1% in men, and 39.9% and 20.4% in women, respectively.^6^ In contrast only 11.5% reported ever having an infection and while most infections are asymptomatic, there is at least a 1% incidence of sexually active women having genital warts at any one time and among women undergoing primary screening, about 8% test positive for oncogenic HPV.^17^ Genital warts are easily transmittable and the high rate of transmission generates a 50% lifetime risk of acquiring genital warts in sexually active individuals and provides additional epidemiologic evidence that infected women may not be aware of having had an HPV infection.^18^ High-risk HPV infections (HR-HPV) are associated with Papanicolaou (PAP) smear change^19^ and recent recommendations incorporate HPV testing into cervical cancer screening. These new screening recommendations incorporating HPV testing means more women will test positive for HPV. This implies that without better communication the gap between having an infection and an awareness of being infected might widen.

Another study reported a prevalence of HR-HPV infection among Mexican American females attending a cervical cancer screening clinic of 13%.^20^ In contrast, our results found that only 1.38% of Mexican American women reported ever being told they had an HPV infection. In comparison, 3.95% White women reported having been told about an HPV infection suggesting a greater infection awareness gap among Mexican American women being told they have HPV and either testing positive or showing clinical manifestations of an HPV infection. Also troubling was the finding that while Black women are disproportionately affected by HPV,^21^ only 1.14% of Black women reported ever being told they had an infection. The disparities between demographic subgroups reporting an infection and epidemiological data highlight that minority women are at greater risk for being unaware of experiencing an infection.

Other studies also identify discrepancies between prevalence and an awareness of being infected with HPV. For example, a 2006 study found that less than half of the women reporting genital warts treatment had heard of HPV, similar to our finding that only 43% of women with genital warts reported ever having had an HPV infection. This helps validate that gaps exist between experiencing a clinically apparent HPV infection and ever being told about an HPV infection suggesting that women health care providers may be missing important “teachable moments” related to educating women about HPV. Research supports that these opportunities can be important motivators for patient behavior change and adherence to treatment.^22^ The persistent failure for most women to connect genital warts to HPV despite awareness campaigns^23^ highlights the need for better communication about the causal connection of HPV infection to genital warts.

The significant burden of HPV in terms of both cost and health underlines the importance of our findings. In the United States, an estimated 10% of the population have an active HPV infection, 4% have an infection that causes cytological abnormalities, and an additional 1% have an infection causing genital warts.^11^ Estimates for annual direct medical costs associated with the prevention and treatment of anogenital warts and cervical HPV-related disease is at least $4 billion and more than 4,000 women succumb annually to cervical cancers. It is critical that women infected with HPV be made aware of their infection and its causal role to PAP smear abnormalities and cervical cancer. Women's awareness of having an HPV infection is associated with increased knowledge, a greater understanding that HPV causes cancer^24^ and links to better adherence with treatment.^25^ Research indicates that women who acknowledge having a previous infection are also more likely to have their children vaccinated.^26^

### Limitations

There are several study limitations. The report of ever having an HPV infection relies on self-report and is subjected to recall bias. However, while women may fail to remember being told, this also suggests poor communication about HPV infection. Also, some women may prefer not to disclose a previous genital infection leading to an under-reporting about previous HPV infection. Since NHANES does not include populations considered at higher risk for HPV such as those institutionalized, incarcerated, and the homeless, this study does not report on these populations. Also, to identify potential gaps between women reporting ever been told they had HPV, epidemiologic findings were used to identify the prevalence of clinically evident infections or infections found by screening rather than chart or case review. While these estimates may not be exact, they consistently demonstrate discordant results between women with positive HPV test results and/or clinically evident genital warts and women reporting they have ever been told they had an HPV infection. Research studying women with positive HPV test results in a clinical setting and/or clinically evident genital warts and reporting being told they have an HPV infection will be helpful to confirm and further characterize potential gaps in awareness of experiencing an HPV infection. A strength of this study is that it represents the first to use a nationally representative sample to provide baseline data about the percentages and characteristics of women reporting about whether they have ever been told they have had an HPV infection.

## Conclusion

Using a national database, this study found that 11.5% of U.S. women reported ever having been told they have had an HPV infection. Epidemiologic data suggest gaps between ever being told of a previous infection and ever being diagnosed with a clinically relevant HPV infection especially among less educated and minority women. Increasing the awareness of having had an HPV infection will likely require combined approaches with strategies targeted to improving provider communication and increasing consumer awareness. Each diagnosed HPV infection represents a “teachable moment” and failing to adequately inform patients represents a missed opportunity to enhance a woman's knowledge of HPV. Despite the consequences of HPV infections, many women remain unaware of HPV and would benefit from being told of an infection.^27–29^

## Abbreviations Used

HPV: human papillomavirus
HR-HPV: high-risk HPV
NHANES: National Health and Nutritional Examination Survey
PAP: Papanicolaous
SD: standard deviation
